# A complex microdeletion 17q12 phenotype in a patient with recurrent *de novo* membranous nephropathy

**DOI:** 10.1186/1471-2369-13-27

**Published:** 2012-05-14

**Authors:** Bernward Hinkes, Karl F Hilgers, Hanno J Bolz, Margarete Goppelt-Struebe, Kerstin Amann, Sandra Nagl, Carsten Bergmann, Wolfgang Rascher, Kai-Uwe Eckardt, Johannes Jacobi

**Affiliations:** 1 Department of Pediatrics, Friedrich-Alexander-University Erlangen-Nürnberg, Erlangen, Germany; 2 Department of Nephrology and Hypertension, Friedrich-Alexander-University Erlangen-Nürnberg, Erlangen, Germany; 3 Center for Human Genetics, Bioscientia, Ingelheim, Germany; 4 Department of Pathology, Friedrich-Alexander-University Erlangen-Nürnberg, Erlangen, Germany; 5 Department of Pediatrics, University of Erlangen-Nürnberg, Loschgestrasse 15, 91054, Erlangen, Germany

**Keywords:** Microdeletion 17q12 syndrome, Mayer-Rokitansky-Kuester-Hauser-Syndrome membranous nephropathy, Nephrotic syndrome, *HNF1β*, *LHX1*

## Abstract

**Background:**

Microdeletions on chromosome 17q12 cause of diverse spectrum of disorders and have only recently been identified as a rare cause of Mayer-Rokitansky-Kuester-Hauser-Syndrome (MRKH), which is characterized by uterus aplasia ± partial/complete vaginal aplasia in females with a regular karyotype. For the first time we report about a patient with a 17q12 microdeletion who is affected by MRKH in combination with a vascular and soft tissue disorder. Repeatedly she suffered from kidney transplant failure caused by consuming membranous nephropathy.

**Case presentation:**

A 38-year-old female patient had been diagnosed with right kidney aplasia, left kidney dysplasia and significantly impaired renal function during infancy. Aged 16 she had to start hemodialysis. Three years later she received her first kidney transplant. Only then she was diagnosed with MRKH. The kidney transplant was lost due to consuming nephrotic syndrome caused by *de novo* membranous nephropathy, as was a second kidney transplant years later*.* In addition, a hyperelasticity syndrome affects the patient with congenital joint laxity, kyphoscoliosis, bilateral hip dysplasia, persistent hypermobility of both elbows, knees and hips. Her clinical picture resembles a combination of traits of a hypermobile and a vascular form of Ehlers-Danlos-Syndrome, but no mutations in the *COL3A1* gene was underlying. Instead, array-based comparative genomic hybridisation (CGH) detected a heterozygous 1.43 Mb deletion on chromosome 17q12 encompassing the two renal developmental genes *HNF1*β and *LHX1.*

**Conclusions:**

Deletions of *HNF1*β have recently drawn significant attention in pediatric nephrology as an important cause of prenatally hyperechogenic kidneys, renal aplasia and renal hypodysplasia. In contrast, membranous nephropathy represents an often-unaccounted cause of nephrotic syndrome in the adult population. A causative connection between theses two conditions has never been postulated, but is suggestive enough in this case to hypothesize it.

## Background

Microdeletions on chromosome 17q12 encompassing the hepatocyte nuclear factor 1β (*HNF1β)* have recently been characterized as an important cause of prenatally hyperechogenic kidneys, renal aplasia and renal hypodysplasia, which represent an important cause of chronic renal failure among children [1,2]. The following case expands the diverse spectrum of disorders associated with 17q12 microdeletions and reveals a hypothetical link with membranous nephropathy (MN), a common and often unaccounted cause of nephrotic syndrome in the adult population.

## Case presentation

**Figure 1:**
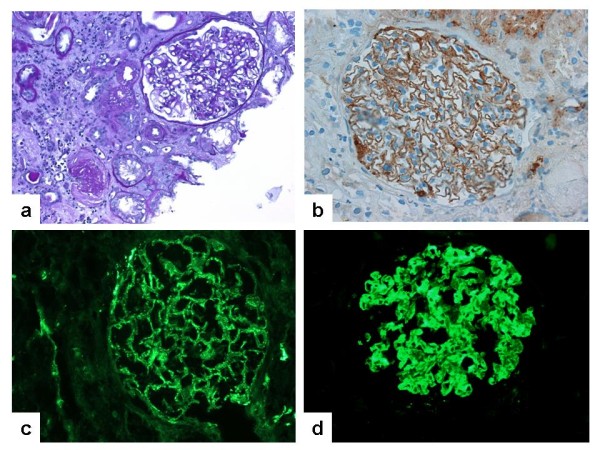
**Percutaneous kidney biopsy four weeks after the second kidney transplantation revealed de-novo MN and moderate tubulo-interstitial changes (a) and granular pseudolinear deposition of immunoglobulin G (IgG) along the epithelial surface of the glomerular basement membrane (GBM)(b).** C3c-deposits along the GBM of the patient were present **(c)**. In reverse, incubation of human kidney samples with serum from the patient showed immunofluorescent staining of the GBM, documenting the presence of anti-human-GBM alloantibodies in the patient **(d)**.

A now 38-year-old female patient was born as the third of four daughters of healthy parents. Her oldest sister remains healthy, while her second oldest sister suffers from idiopathic focal epilepsy and her youngest sister from Prader-Willi-Syndrome caused by a maternal parental disomy on chromosome 15. Following a regular pregnancy our patient first presented with a pyelonephritis at the age of four months. Right kidney aplasia and a significantly impaired renal function (serum creatinine 1.9 mg/dl, urea 83 mg/dl) were then noted, already indicating dysplasia of the left kidney. Her female external genitalia showed no anomalies and cystoscopy documented the presence of bilateral ureters. Over the following years chronic renal failure progressed as recurrent urinary tract infections occurred. Finally, she was referred to a pediatric nephrology unit at the age of 10 years and started hemodialysis six years later with a serum creatinine of 6 mg/dl. Aged 19 she received her first kidney transplant without any complications. As primary amenorrhea had persisted while pubertal development was commensurate with age, a diagnostic laparoscopy was undertaken and revealed a rudimentary uterus and regular ovaries. On the basis of a normal 46/XX karyotype the diagnosis of atypical Mayer-Rokitansky-Kuester-Hauser-Syndrome (i.e. MRKH Type II) was made. Aged 28 her first kidney transplant was lost due to consuming nephrotic syndrome caused by biopsy-proven *de novo* MN*.* At the age of 36 a second kidney transplantation was performed. Despite thymoglobulin induction and an intensified triple immunosuppressive therapy (tacrolimus, mycophenolate mofetil, prednisolone), under which she developed new onset diabetes mellitus after transplantation (NODAT), MN reoccurred within only four weeks after transplantation. Testing for secondary causes of MN such as hepatitis, HIV and antibodies against the phospholipase-A2 receptor (PLA2R) was negative [3]. All attempts to reverse MN including high dose steroids, immunoglobulin, plasma- and immunoabsorption, anti-CD20-antibodies (Rituximab) and proteasome inhibitor (Bortezomib) failed. Finally, the transplant had to be explanted despite remaining renal function (creatinine 2.4 mg/dl) due to the severity of nephrotic syndrome (albuminuria ≤ 15 g/g creatinine; Figure 1).

**Figure 2:**
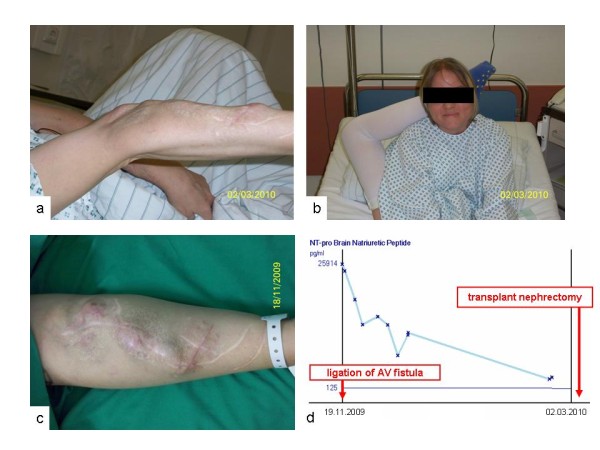
**Vascular and soft tissue disorder of the patient with hypermobility of both elbows (a) and hips (b).** Hyperdynamic arterio-venous fistulas on both arms **(c)** led to tricuspid valve insufficiency and required ligation, which resulted in a rapid decline of the significantly raised brain natriuretic peptide (BNP) **(d)**.

In addition to her urogenital anomalies our patient is affected by a hyperelasticity syndrome with congenital joint laxity, kyphoscoliosis, and bilateral hip dysplasia. She shows persistent hypermobility of both elbows and knees and is easily able to place her feet behind her head, all of which are very unusual findings in a longstanding dialysis patient (Figure 2a-c)*.* Her Beighton score is currently 8/9. As a result of dilating hyperelastic vascular walls arterio-venous fistulas on both arms rapidly developed hyperdynamic flows. Repeated surgical banding of the fistulas accomplished only a temporary improvement, so that vascular access was ligated shortly after transplantation to control right heart failure with tricuspid valve insufficiency (Figure 2d)*.*

In summary, this clinical picture resembles a mixture between a hypermobile (EDS3) and a vascular (EDS4) form of Ehlers-Danlos-Syndrome (EDS). Mutation analysis of the collagen type 3 alpha 1 gene (*COL3A1)*, which is mutated in EDS4 and rarely in EDS3, was negative. We did not analyze *tenascin-X* (*TNXB*), another rare cause of EDS3, because characteristic hyperelastic skin changes were missing in our case. Mutations in the *FBN1* gene, which is mutated in Marfan syndrome, had been excluded previously. As a next step a skin biopsy was taken for electron microscopy. It revealed a soft tissue disorder with loosely packed collagen fibres. The picture resembled findings in EDS4, without meeting the obligatory criteria of this disease, and was not suggestive of any other form of EDS.

**Figure 3:**
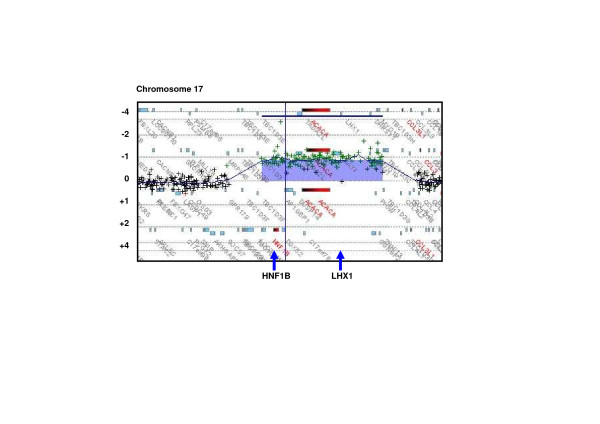
**The heterozygous 1.43 Mb deletion arr 17q12(34.817.222-36.249.059)x1** (ISCN 2009, GRCh37/hg19) containing the sixteen RefSeq genes *ZNHIT3, MYO19, PIGW, GGNBP2, DHRS11, MRM1, LHX1, AATF, ACACA, C17orf78, TADA2L, DUSP14, AP1GBP1, DDX52, HNF1β, LOC2841100*, as detected by array-based comparative genomic hybridisation (Array-CGH, Oligo-Microarray Human Genome 244A; Agilent Technologies).

We therefore performed an array-based comparative genomic hybridisation (CGH) and detected a heterozygous 1.43 Mb deletion on chromosome 17q12 (Figure 3). This deletion contained sixteen RefSeq genes and was absent in both the father and the mother of our patient, consistent with a *de novo* copy number variant (CNV).

## Conclusions

Mayer-Rokitansky-Kuester-Hauser (MRKH) is a quite common inherited condition (1:4.000-5.000) characterized by uterus aplasia ± partial/complete vaginal aplasia in females with a regular karyotype XX/46 and physiological ovarial function [4-6]. Microdeletions of chromosome 17q12 including *HNF1β,* such as the one detected in this case have recently been identified as a rare cause of MRKH [4-6]. In a series of only five published cases, three women presented without (MRKH type I/typical) and two with kidney affection (MRKH type II/atypical). This finding reflects the highly variable clinical picture of even identical 17q12 microdeletions, reaching from diabetes (MODY5 or NODAT, as observed in our patient), to pancreas and liver anomalies, epilepsy, sensorineural hearing loss, cognitive impairment, autism and even schizophrenia [7-10]. Our patient has no neurological impairments and works as a teacher. Nevertheless, in addition to previously reported cases, she is affected by a soft tissue disorder with joint hypermobility and vascular hyperelasticity resembling a picture with features of both the hypermobile (EDS3) and the vascular (EDS4) form Ehlers-Danlos-Syndrome. We observed structural collagen changes of the skin in electron and light microscopy and excluded mutations in *COL3A1*. A causative genetic defect in another unknown locus is thereby not excluded. However, joint laxity has been described in selected individuals with deletions on 17q12 (DECIPHER patients 248627, 249204) and could result from the microdeletion itself.

Following kidney transplantation our patient repeatedly developed MN, a major cause of nephrotic syndrome in adults. We excluded secondary causes of MN and detected no *PLA2R* autoantibodies, which are found in approximately 75% of all cases of “idiopathic” MN in the adult population [3]. The identification of further glomerular components as targets for autoantibodies in MN has been suggested [3]. Our patient developed MN and consuming nephrotic syndrome years after her first, but only weeks after her second kidney transplantation. This clinical course may reflect a boostered alloimmune response against the kidney transplants, or alternatively a boostered response against a physiological glomerular epitope, which had not been expressed in the patient’s native kidney. Consistent with the second assumption, incubation of renal tissue specimens from healthy human controls with the patients’ serum resulted in granular deposits of immunoglobulin G (IgG) along the surface of the glomerular basement membrane (Figure 1d). Staining of respective mouse and rat kidney samples was negative. No candidate gene encoding a structural component of the glomerular filtration barrier as a hypothetical antibody target is located within the microdeletion on 17q12. Still, it is remarkable that the deletion includes two transcription factors, which are important in kidney development. *HNF1β* and *LHX1* are physically adjacent genes on 17q12. Their gene products are involved in one signaling pathway in which *HNF1β* directly activates the promotor of *LHX1*[11]. *LHX1* is a major and dose-dependent regulator of various steps of renal and urogenital development. In the kidney *LHX1* is required for epithelial tubular genesis and for podocyte development, including the crucial morphogenic step of the glomerulus from a comma to an S-shape stage and consecutively for the development of the glomerular filtration barrier [12,13]. The significance of *LHX1* in human disease remains an open question and it will be interesting to observe if eventually a connection between alterations of *LHX1* and cases of membranous nephropathy such as ours can be demonstrated.

Above all, our case illustrates how recent molecular findings in inborn orphan disease may permit the diagnosis for complex phenotypes in adult patients - a circumstance which was truly appreciated by our patient 38 years after her first clinical presentation.

## Consent

Written informed consent was obtained from the patient for publication of this Case Report and any accompanying images. A copy of the written consent is available for review by the Series Editor of this journal.

## Abbreviations

CNV, Copy number variants; COL3A1, Collagen type III, alpha 1; EDS, Ehlers-danlos-syndrome; FBN1, Fibrillin 1 gene; GBM, Glomerular basement membrane; HNF1β, Hepatocyte nuclear factor-1β gene; LHX1, Homeobox transcription factor 1 gene; MN, Membranous nephropathy; MRKH, Mayer-rokitansky-kuester-hauser-syndrome; NODAT, New onset diabetes mellitus after transplantation; NT-pro BNP, N-terminal pro brain natriuretic peptide; PLA2R, Phospholipase A2 receptor.

## Competing interest

The author(s) declare that they have no competing interest.

## Authors' contributions

BH wrote and revised the manuscript, coordinated the studies for this manuscript. JJ was the treating physician and substantially supported the writting of this manuscript. KA carried out the nephropathologic studies. HB, SN, CB carried out the molecular genetic studies. KH, MGS, WR, KUE supported this work by providing critique of succesive drafts of the manuscript. All authors read and approved the final manuscript.

## Pre-publication history

The pre-publication history for this paper can be accessed here:

http://www.biomedcentral.com/1471-2369/13/27/prepub
